# Initial Validation of the MAKE Framework: A Comprehensive Instrument for Evaluating the Efficacy of Game-Based Learning and Gamification in Adolescent Sexual Health Literacy

**DOI:** 10.5334/aogh.1110

**Published:** 2019-02-28

**Authors:** Hussein Haruna, Xiao Hu, Samuel Kai Wah Chu, Robin R. Mellecker

**Affiliations:** 1 Faculty of Education, The University of Hong Kong, Pokfulam Road, HK

## Abstract

**Objectives:**

When evaluating the effectiveness of a method for instructing adolescents in sexual health literacy, it is essential to consider how the method **m**otivates learning, promotes a change of **a**ttitude, increases **k**nowledge gain, and **e**ngages students (MAKE). This article reports on the development and validation of a unified, comprehensive framework for evaluating the efficacy of games in teaching sexual health behaviors for curbing unhealthy sexual outcomes to secondary school adolescents in low resource settings.

**Methods:**

The initial validation of the MAKE framework was administered to 120 students using quantitative data collection and analysis. It was then subjected to factor analysis tests to investigate the items’ structure, and Cronbach’s alpha was applied to measure the scale reliability using SPSS Version 24.

**Results:**

Data analyses demonstrate that the MAKE framework is a comprehensive instrument to evaluate teaching methods with four powerful constructs, each of which has two to four components. For each construct, the following data were obtained: for motivation, standardized alpha = 0.92, Kaiser-Meyer-Olkin (KMO) = 0.88, and p = 0.001; for attitude, standardized Cronbach’s alpha = 0.90, KMO = 0.88, and p = 0.001; for knowledge, standardized alpha = 0.92, KMO = 0.86, and p = 0.001; and finally, for engagement, standardized alpha = 0.90, KMO = 0.87, and p = 0.001. Cronbach’s alpha for each component was above the cut-off point (0.65).

**Conclusions:**

This study shows that the MAKE framework is a satisfactory instrument for assessing the efficacy of teaching methods for sexual health literacy in a variety of teaching environments. The method may also have value for assessing the effectiveness of other methods in adolescent sexual health education.

## Introduction

Evaluating the efficacy of teaching methods is necessary to determine the impact of sexual health literacy interventions among adolescents [1]. Ensuring the reliability and validity of the developed or adapted measures [2] used in evaluation is thus important. Validation of health related instruments has been reported to very essential [3,4,5]. Thus, in this study, we combined elements of other studies (see Appendix A) to create a framework, named MAKE, for assessing success in teaching and learning in terms of knowledge acquisition. MAKE is an abbreviation for teachers’ **m**otivating learning, promoting a change of **a**ttitude, enhance **k**nowledge acquisition, and **e**ngaging students. We used this framework to evaluate the effectiveness of designed and developed game-based learning (GBL), gamification instructional interventions in comparison with existing traditional teaching approach used in sexual health education. Each of the four MAKE constructs is crucial in assessing the effectiveness of sexual health education programs. In previous studies, the MAKE constructs were used separately. In this study, the constructs were integrated and validated as an instrument to evaluate teaching strategies using game-based learning and gamification.

Each of the four MAKE constructs has been used in prior research to evaluate sexual health education. However, to our understanding, none of the study has reported to use all of them in conjunction. There is thus a lack of sufficient empirical research to support the development, integration, validity, reliability, and applicability of a comprehensive MAKE framework for evaluating teaching methods in adolescents’ sexual health education. The current study develops and evaluates the comprehensive MAKE framework’s reliability and validity when examining game-based learning and gamification digital health interventions for improving sexual health education in comparison with traditional teaching in secondary schools among adolescent students. The MAKE framework was developed according to various theories and models in the educational and health promotional research literature. Each of the four constructs is elaborated below.

### Motivation

Motivation refers to “what experiences or goals [people] will approach or avoid and the degree of effort they will exert in that respect” [6 (p3)]. In education, motivation is “a student’s willingness, need, desire and compulsion to participate in, and be successful in, the learning process,” and the goal is “to increase the factors that move a student toward becoming more involved in the class and the subject matter” [6 (p3)]. The ARCS model of motivation evaluates the efficacy of teaching and learning with respect to four components that motivate learning: attention, relevance, confidence, and satisfaction [7]. In recent years, “game-based learning” has been used in conjunction with the ARCS model [8]. In the present study, ARCS is used to operationalize motivation when evaluating the effectiveness of gamified learning platforms (game-based learning and gamification) in supporting adolescent learning and acquire healthy sexual knowledge and skills for responsible well-being sexual behavior practices.

### Attitude

Attitude has been used in research [9,10,11,12,13] to measure the effectiveness of sexual health education. Attitude refers to people’s feelings or judgements, which can be either positive or negative [14]. Evaluating attitude is crucial because, like motivation, it is linked to human behavior. In most cases, a person’s behavior is affected by his or her attitudes. There are three components of attitude: affect, cognition, and behavior [14]. Attitude denotes what a person believes and feels about something and how his or her beliefs and feelings affect his or her behavior [15]. Sexual health education programs are more likely to be successful if learners’ attitudes are taken into account [16]. The present study evaluates learners’ perceptions of the effectiveness of game-based learning and gamification when compared with traditional methods of sexual health education using affective attitude and cognitive attitude. The study will examine students’ perception and how they felt about the three teaching methods used. Furthermore, it evaluates the impact of the three teaching methods on improving students’ positive attitudes towards healthy sexual behavior practices.

### Knowledge

In any sexual health education program, the intention is to improve and help acquisition of knowledge of sexual health that allows adolescents to make informed decisions, as they will better understand the consequences of underage sexual activity [17]. As such, it is important to evaluate whether teaching methods increase knowledge about sexual health. This study evaluates students’ views on the efficacy of game-based learning and gamification digital health interventions when compared with traditional teaching models in enhancing sexual health knowledge acquisition.

### Engagement

In education, engagement refers to the maximal connection with a student in the learning process [18]. Like attitude, engagement comprises three components: cognition, emotion, and behavior [19]. Numerous observable characteristics can be used to evaluate learners’ engagement, including body language, consistency, participation, confidence, fun, interest, and excitement. Non-observable characteristics that need to be evaluated include individual attention, clarity of learning, meaningfulness of work, rigor in thinking, and performance orientation. However, these varied characteristics can be organized into three main categories that act as a catalyst for learners to foster and develop a sense of commitment and belonging in the learning process: enjoyment, interest, and challenge [20]. However, this study examines two types of engagements: emotional engagement and cognitive engagement.

## Methods

### Overview of the research procedure

Game-based learning and gamification digital health interventions were designed and developed to deliver sexual health knowledge among adolescent students in Tanzania during their sexual health education classes. Game-based learning is a kind of game play and game content that enhance knowledge acquisition with a purpose to achieve a defined teaching and learning outcomes [21]. In this context, “gamification” is the application of game elements or game mechanics (such as rewards, challenges, competitions, points, etc.) into non-gaming context for the purpose of engaging, motivating, promoting to learn, and solving a certain problem. Even prior research suggested gamification could be applicable in sexual health education among adolescents [22]. This study employed different game elements readily added on the Moodle platform. Various stakeholders were engaged through a participatory design research [12,23] and community engagement approach [24] to develop and suggest the use of the MAKE evaluation framework in the study setting, including: a game designer, computer and information sciences specialists, secondary school teachers, sexual and reproductive health specialists, students, and members of the community.

The game-based learning and gamification digital health interventions were designed and incorporated into learning activities. After experiencing the game-based learning and gamification interventions, students attended a series of classes that covered five topics relevant to adolescent sexual health: personal hygiene and good manners; sexual responsibility and decision making; peer pressure; sexually transmitted infections (STI) prevention, including Human Immunodeficiency Virus (HIV)/Acquired Immunodeficiency Syndrome (AIDS); and dealing with harmful practices and sexual violence. Using quasi-experiment research design, students from three classes were randomly assigned to each condition based on the class they were in. Students were randomly divided into three groups based on three teaching conditions: experimental condition students assigned to the game-based learning and gamification digital health interventions, and traditional teaching students who were assigned to a control condition. The series of learning lasted for five-weeks, whereby each topic lasted for 40 minutes per week. Students were not aware of these or other conditions. The control conditions were in a regular classroom while the experimental conditions were in computer labs.

### Participants and setting

Participants were secondary school students enrolled in one secondary school in Dar Es Salaam Tanzania. They were Form One (equivalent to year seven in the British educational system) in their first year of ordinary/lower secondary education. They were aged 11 to 15 years. A total of 120 students were recruited. They were from three classes with the same educational status. Each condition had 40 students. Ethical approval for the study was obtained from the Human Research Ethics Committee at the University of Hong Kong and the National Institute for Medical Research in Tanzania.

### Measure

The students received a questionnaire based on the MAKE framework that included questions about the efficacy of the three teaching methods. Assessment tools were adapted [25,26,27] and were modified to align with the game-based learning and gamification digital health learning methods. The motivation construct had 16 items and the three remaining constructs (attitude, knowledge, and engagement) each had 10 items. Each item was rated on a 5-point Likert scale from 1 (strongly disagree) to 5 (strongly agree). The goal was to validate the MAKE instrument to evaluate the instructional approach for motivating learning, promoting a change of attitude, enhancing knowledge, and engaging students during the learning process. Students were given 15 minutes to complete self-rating questionnaire (See Appendix F).

### Data analysis

The collected quantitative data were analyzed using IBM SPSS Statistics Version 24. Factor analysis tests were used to group related scale items and measurements into more manageable components. The purpose was to group related items or variables while developing scales and measures to make it easier to identify the underlying structure of items. Factor analyses were conducted for all of the survey items to evaluate their applicability for each MAKE evaluation construct. Consequently, the measuring instruments ensured and increased validity [28]. The SPSS data analysis tool was also used to test the scale reliability for this study. Descriptive statistics tests were conducted to generate mean, median, and standard deviation variables for each of the four constructs unified herein referred to MAKE evaluation framework.

## Results

### Demographic information for participants

A little more than half (52.5%) of the students were male, while the rest (47.5%) were female. The ages of respondents were 15 years (42.5%), 14 years (32.5%), 13 years (17.5%), 12 years (7.5%), and 11 years (0%). The mean age for boys was 14.22 years, whereas the mean age for girls was 13.96 (0.96) years. More than 66.7% of respondents indicated that they lived at home with both a father and a mother, 17.5% with a mother only, 10% with a guardian, and 5.8% with a father only. All participants were almost equal in terms of socioeconomic status, as measured by the education level, occupation, and family living status of parents and guardians.

### General Factor analysis and scale reliability

Details for each MAKE construct, the general results of the factor analysis and scale reliability for each MAKE construct is presented in Table 1. Construct items are listed in Appendix B.

**Table 1:** Factor Analysis and Scale Reliability for MAKE Evaluation Tool (N = 120).

Constructs	Components	No. of items	Alpha	Standardised Alpha	KMO	P value

Motivation	Attitude	4	0.92	0.92	0.88	0.001*
	Relevance	4	0.93				
	Confidence	4	0.90				
	Satisfaction	4	0.85				
Attitude	Affective	5	0.91	0.90	0.88	0.001*
	Cognitive	5	0.89				
Knowledge	Importance	4	0.93	0.92	0.86	0.001*
	Effectiveness	3	0.93				
	Application	3	0.89				
Engagement	Emotional	6	0.91	0.90	0.87	0.001*
	Cognitive	4	0.88				

### Specific Factor analysis and scale reliability

Specifically, this section will present factor analysis and scale reliability for each construct of the MAKE framework.

### Motivation construct

#### Factor analysis

This study adapted 16 items of the attention, relevance, confidence, and satisfaction (ACRS) model from the Instructional Materials Motivational Survey (IMMS) to serve as measures of the motivation construct. These IMMS items were validated in prior study [28] and were used to evaluate the effectiveness of game-based learning in motivating learners [29]. Initially, the IMMS contained 36 items to measure the ARCS components on a 5-point Likert scale [30], ranging from strongly disagree (1) to strongly agree (5). In this study, factor analysis was conducted to verify the 16 adapted items. The Kaiser-Meyer-Olkin (KMO) measure of sampling adequacy was 0.87, and Bartlett’s test of sphericity was significant (p = 0.001). Thus, the factor analysis was strong. The rotated component matrix grouped 16 motivation items into 4 subcomponents for each component of the ARCS model used in this study (See Appendix B).

#### Scale reliability

Empirical data suggest that the motivation construct has good scale reliability. For example, other scholars [26] used 20 items of the ARCS model to evaluate learners’ motivation in digital game-based learning and reported a Cronbach’s alpha value of 0.88. In the current study, the overall scale reliability statistics indicated that Cronbach’s alpha was 0.92, and the same Cronbach’s alpha value was based on standardized items, namely, 0.92 (n = 120 on 16 items). This represents a strong reliability of scale. Furthermore, the subscale reliability for each component was as follows: for attention, Cronbach’s alpha = 0.92, with a mean of 15.19 (4.601); for relevance, Cronbach’s alpha = 0.93, with a mean of 15.73 (3.90); for confidence, Cronbach’s alpha = 0.90, with a mean of 16.95 (3.39); and finally, for satisfaction, Cronbach’s alpha = 0.85, with a mean of 17.03 (2.76) (see Appendix B).

### Attitude component

#### Factor analysis

The attitude survey instrument was adapted from prior study [27] with the intention of evaluating students’ attitude towards the teaching methods used in sexual health education. Furthermore, the instrument examined whether students’ attitudes toward irresponsible sexual behavior could be changed. This study performed factor analysis to verify 10 items used to measure attitude. The KMO value was 0.88, and Bartlett’s test of sphericity reached statistical significance (p = 0.001), thus supporting the factor analysis. A rotated component matrix grouped 10 attitude items into 2 groups: affective attitude (5 items) and cognitive attitude (5 items) (see Appendix C).

#### Scale reliability

Wernersbach (2013) reported Cronbach’s alpha as 0.77 for sexual health attitudes. By using a 5-point Likert scale, ranging from 1 (strongly disagree) to 5 (strongly agree), the overall reliability of the scale for this study achieved a Cronbach’s alpha value of 0.90, and Cronbach’s alpha based on standardized items was 0.91, indicating good reliability for the scale. For the subscale reliability of affective attitude, Cronbach’s alpha = 0.91, with a mean of 21.93 (4.15), while for cognitive attitude, Cronbach’s alpha = 0.89, with a mean of 21.72 (4.12).

### Knowledge component

#### Factor analysis

Ten knowledge statements evaluated the teaching methods using a 5-point Likert scale, ranging from 1 (strongly disagree) to 5 (strongly agree). A factor analysis test was performed to verify knowledge statements. The KMO value was 0.86 and Bartlett’s test of sphericity was p = 0.001, thus strongly supporting the analysis. The rotated component matrix categorized 10 knowledge items into 3 groups: importance of knowledge (4 items), effectiveness of knowledge (3 items), and application of knowledge (3 items). Details for factor analysis are provided in Appendix D.

#### Scale reliability

For the overall reliability of the scale, Cronbach’s alpha was 0.92, and Cronbach’s alpha based on the 10 standardized items was 0.92, thus demonstrating good reliability for the scale. For the subscale reliability for importance of knowledge, Cronbach’s alpha = 0.93, with a mean of 17.01 (4.18); for effectiveness of knowledge, Cronbach’s alpha = 0.93, with a mean of 12.48 (3.33); and finally, for application of knowledge, Cronbach’s alpha = 0.89, with a mean of 13.58 (2.04) (see Appendix D).

### Engagement component

#### Factor analysis

Ten statements were used to evaluate the teaching methods according to student engagement using a 5-point Likert scale, ranging from 1 (strongly disagree) to 5 (strongly agree). A factor analysis test was conducted to verify engagement statements. The KMO value was 0.87, and Bartlett’s test of sphericity was p = 0.001, strongly supporting the analysis. The rotated component matrix categorized engagement statements into two components: emotional engagement (six items) and cognitive engagement (four items).

#### Scale Reliability

The scale reliability test was conducted for all 10 engagement variables. For the overall reliability of the scale, Cronbach’s alpha = 0.90, and Cronbach’s alpha based on all 10 standardized variables = 90, revealing a strong reliability for the scale. For the subscale reliability for emotional engagement, Cronbach’s alpha = 0.91, with a mean of 24.12 (6.458); and for cognitive engagement, Cronbach’s alpha = 0.88, with a mean of 16.38 (3.870).

## Discussion

The findings confirmed prior research [28], which indicated that research instruments should be validated and surveyed items should be modified to meet the requirements of the study setting. This previous research provided a crucial step in evaluating the validity and reliability of the MAKE framework in relation to examining the effective of the teaching methods. Each of the MAKE constructs has been found to be independently valid in measuring student learning outcomes which had never been validated in conjunction.

Motivation constructs (ARCS) were previously found to be valid for evaluating the efficacy of different teaching methods [29]. This study adapted only 16 motivational items [28,29] which were determined to be relevant to the study context. Of these 16, all were retained, and the rotated component matrix grouped 4 items for each of the components of the ARCS motivation model. In the study [28], the adapted motivational ARCS model was found to be effective in evaluating game-based learning environments. In that study, 16 of the original 36 items were excluded; the 20 remaining items met the validation criteria and were thus retained. By contrast, this study adapted 16 items, all of which were retained [29]. This discrepancy can be attributed to the following factors: The research [28] focused on a computer-based tutorial known as M-Tutor (which was different from games learning); participants were college students (not secondary school students); and/or the course was in engineering (not sexual health behaviour course). In addition, the two studies differed in terms of context: one was conducted in a developed country, while the current study was conducted in a developing country where resources were limited.

Attitude items are important in evaluating teaching methods, as they are linked to human behavior: they indicate whether participants had positive or negative feelings toward a teaching method and if the method helped them change their attitude toward irresponsible sexual health behavior. There were 10 attitude items used to evaluate teaching methods in this study, and all were retained. The rotated component matrix grouped attitudes into two constructs: affective and cognitive. Each had five measurements. Behavioral attitudes were excluded in the study because assessing behavior requires a longitudinal study to observe changes in a participant’s behavior. The attitude items were adapted from prior research [27], for which the reported Cronbach’s alpha value was 0.77 (above the preferred 0.70) [31]. Cronbach’s alpha for the current study was 0.90, thus outperforming the prior research. Unlike [27], who did not report a factor analysis in his validation of attitude instruments, the current study’s adapted instrument showed excellent validity and was satisfactory for evaluating teaching methods.

Beyond attitude, a teaching method’s effectiveness can also be evaluated in terms of learners’ knowledge gain. In this study, knowledge gain instruments evaluated learners’ perceptions of the teaching methods used. Ten knowledge statements were tested to verify the validity of the measures. After conducting factor analysis and scale reliability tests, the results indicated that all 10 items met the standard for evaluating sexual health education teaching methods. The rotated component matrix clumped these items into three categories: one with four items and the remaining two with three items each. These were as follows: the importance of knowledge, the effectiveness of knowledge, and the application of knowledge. This study thus considered the knowledge assessment construct to be strong enough to evaluate students’ perception towards sexual health education in its context. However, more validation is required if it is to be used in other settings.

Finally, an initial validation of the engagement instruments was carried out to assess their suitability for evaluating game-based learning and gamification digital health interventions in sexual health education relative to traditional methods. The findings confirmed that the adapted engagement items were satisfactory for examining teaching methods. However, the results differed from previous research [25], which did not report an empirical approach to validate the instruments. Instead, the study [25] reported the use of other people, who were not involved in the research project, as evidence of the reliability of the collected data. In this study, the instruments used to evaluate teaching methods and student engagement appeared to be successful, having met the required standards for validity and reliability.

## Conclusion and Further Study

This study validates research instruments adapted from various sources and proposes the MAKE framework as an instrument for assessing the effectiveness of sexual health education using game-based learning and gamification digital health interventions. Unlike numerous studies that evaluated sexual health education using the constructs of MAKE independently, this study integrates the constructs and verifies their validity and reliability. Future research should use the MAKE framework to evaluate game-based learning and gamification digital health interventions instructional approaches for enhancing sexual health knowledge relative to traditional teaching approaches. This study shows that the MAKE framework is a satisfactory instrument for assessing the efficacy of teaching methods in a variety of interactive teaching environments that intents to support students’ knowledge acquisition and its application in a real-world setting.

Because this research project was conducted in three iterations, the results of the first round that used MAKE framework is written in another article. However, the forthcoming two rounds of this research project will use MAKE to evaluate and compare the three teaching methods, while assessing its validity and reliability. The MAKE framework is relatively straightforward to use; researchers, school teachers and other key stakeholders who working in health promotion and education for improve health outcomes can use it to assess sexual health education. However, this study’s results are limited in terms of settings; its research was limited to Tanzanian secondary schools. As such, the findings cannot be generalized to other settings. Further research is required to validate the MAKE framework in other settings globally.

## Additional Files

The additional files for this article can be found as follows:

10.5334/aogh.1110.s1 Appendix A. Literature Evaluating One or More MAKE Evaluation Domain.

10.5334/aogh.1110.s1 Appendix B. Rotated Component Matrix for the Motivation.

10.5334/aogh.1110.s1 Appendix C. Rotated Component Matrix for Attitude.

10.5334/aogh.1110.s1 Appendix D. Rotated Component Matrix for the Knowledge.

10.5334/aogh.1110.s1 Appendix E. Rotated Component Matrix for the Engagement.

10.5334/aogh.1110.s1 Appendix F. MAKE Evaluation: Survey Questionnaire.
