# Supplementary material for: Initial Validation of the MAKE Framework: A Comprehensive Instrument for Evaluating the Efficacy of Game-Based Learning and Gamification in Adolescent Sexual Health Literacy
Source: Ann Glob Health. 2019 Feb 28;85(1):19. doi: 10.5334/aogh.1110 (PMC7052344; doi:10.5334/aogh.1110)
Supplement: Appendix A. — Literature Evaluating One or More MAKE Evaluation Domain. [file agh-85-1-1110-s1.pdf]

Appendix A:

Literature Evaluating One or More MAKE Evaluation Domain

Author	Research Title	Evaluation Domain
[9]	'It's like you're actually playing yourself': Development and preliminary evaluation of 'Green Acres High', a serious game-based primary intervention to combat adolescent dating violence.	Attitude
[32]	Game-Based Intervention: A technical tool for social workers to combat adolescent dating violence	Engagement
[10]	Adolescents' pregnancy prevention by health education computer games: Computer-assisted instruction of knowledge and attitude.	Attitude<br><br>Knowledge
[33]	Serious game for relationships and sex education: Promoting discourse on pressure and coercion in adolescent relationships.	Engagement
[11]	Student and teacher perceptions of game plan: A middle-level sex education programme.	Attitude
[34]	Computer-assisted instruction: An effective instructional method for HIV prevention education.	Motivation<br><br>Knowledge
[35]	Evaluation ability assessment of adolescent pregnancy prevention and sexual health program 'Be Proud! Be Responsible! In New York State.	Engagement
[12]	Promoting sex education among teenagers through an interactive game: Reasons for success and implications.	Attitude<br><br>Knowledge
[13]	Measuring the effectiveness of teaching sex education in Nepalese secondary schools: An outcome from a randomized ccontrolled trial.	Attitude<br>Engagement<br>Knowledge

Appendix B:

Rotated Component Matrix for the Motivation

Component	Motivation statement	1	2	3	4
Attention	There was something interesting at the beginning of the instructional method that got my attention.	0.846			
	The teaching approach used is eye-catching.	0.721			
	The quality of the activity in the teaching method holds my attention.	0.703			
	The design of the teaching method looks appealing.	0.699			
Relevance	I could relate the content taught in this method to things I have thought about in my own future life.		0.857		
	The content taught through this approach will be useful during my adolescent period.		0.854		
	The content and instructional style convey the impression that the course is worth knowing.		0.832		
	The content in the teaching approach will be useful to me.		0.771		
Confidence	I could not really understand quite a bit of the material taught using this method.			0.881	
	The exercises in this teaching approach were too easy.			0.856	
	The good organization of the content helped me be confident that I would learn better with this approach.			0.839	
	The teaching approach was more difficult to understand than I would like it to be.			0.820	
Satisfaction	I really enjoyed learning with this teaching method.				0.827
	It was a pleasure to learn sexual health behavior through this pedagogy.				0.812
	Completing the exercise in this teaching method gave me a satisfying feeling of accomplishment.				0.752
	I learned some things that were surprising or unexpected with this teaching method.				0.730

Appendix C:

Rotated Component Matrix for Attitude

Component	Attitude statement	1	2
Affective Attitude	The instructional approach increases my participation in sexual health education.	0.900	
	I feel happy to be taught sexual health education through this teaching method.	0.892	
	The teaching method used makes sexual health education more interesting.	0.890	
	The teaching method I experienced is appropriate for the delivery of sexual health skills.	0.885	
	The teaching method used is ideal for sexual health education.	0.597	
Cognitive Attitude	The teaching method I experienced enhanced my understanding of sexual health behaviour issues.		0.859
	The instructional approach explained the sexual health learning materials very well.		0.858
	I found the instructional method worthwhile in the sexual health course.		0.854
	The method of instruction used in the sexual health course I attended was interesting.		0.803
	The teaching method used aroused interest in sexual health education programs.		0.708

Appendix D:

Rotated Component Matrix for the Knowledge

Component	Knowledge statement	1	2	3
Importance of knowledge	The teaching approach enhanced my knowledge about STDs, STIs and HIV/AIDS.	0.881		
	I gained knowledge about reproductive health through this teaching method.	0.869		
	I gained knowledge about sexual decision-making through this method of instruction.	0.825		
	The instructional method used helped me learn about good manners and personal hygiene.	0.746		
Application of knowledge	I will apply the sexual coercion and assault knowledge taught through this approach.		0.895	
	The topic of responsible sexual behavioural practices seems very relevant as taught by this approach.		0.887	
	The method of teaching helped me learn about dealing with peer pressure during adolescence.		0.793	
Effectiveness of knowledge	The method of instruction is very active and it helped my understanding of the importance of abstinence.			0.870
	The teaching approach is very effective because it extended my existing understanding of the course.			0.868
	This teaching method is very effective in imparting knowledge in sexual health education programmes.			0.734

Appendix E:

Rotated Component Matrix for the Engagement

**Component**	**Engagement statement**	**1**	**2**
Emotional engagement	In the teaching method I attended, it was very easy to understand the instructional content.	0.928	
	I have been effective in this course because the method of instruction was engaging.	0.924	
	The teaching method used facilitated my active participation in the subject taught.	0.923	
	The method of instruction used caught my attention during the course.	0.869	
	This method allowed my expression of thoughtful ideas relevant to the course.	0.823	
	The instructional approach used during the course made me interested.	0.778	
Cognitive engagement	I demonstrated my interest and enthusiasm as well as use of positive humour during the course.		0.822
	This teaching method is relevant for engaging students in the sexual health education course.		0.809
	The teaching strategy enhanced my engagement in the course.		0.779
	I focused on the learning activities given in this teaching approach.		0.742

Appendix F:

MAKE Evaluation: Survey Questionnaire

Measure	SD	D	N	A	SA
**MOTIVATION: Attention Statement**					
There was something interesting at the beginning of the instructional method that got my attention.	☐	☐	☐	☐	☐
The teaching approach used is eye catching.	☐	☐	☐	☐	☐
The quality of activity in the teaching method holds my attention.	☐	☐	☐	☐	☐
The design of the teaching method looks appealing.	☐	☐	☐	☐	☐
**MOTIVATION: Relevance Statement**					
I could relate the content taught through this method to things I have thought about in my own future life.	☐	☐	☐	☐	☐
The content taught through this approach will be useful during my adolescent period.	☐	☐	☐	☐	☐
The content and instructional style convey the impression that the course is worth knowing.	☐	☐	☐	☐	☐
The content in the teaching approach will be useful to me.	☐	☐	☐	☐	☐
**MOTIVATION: Confidence Statement**					
I could really understand quite easily of the material taught through this teaching method.	☐	☐	☐	☐	☐
The exercises in this teaching approach were too easy.	☐	☐	☐	☐	☐
The good organization of the content helped me be confident that I would learn better in this approach.	☐	☐	☐	☐	☐
The teaching approach was simpler to understand than I would like for it to be.	☐	☐	☐	☐	☐
**MOTIVATION: Satisfaction Statement**					
I really enjoyed learning with this teaching method.	☐	☐	☐	☐	☐
It was a pleasure to learn sexual health behaviors through this pedagogy.	☐	☐	☐	☐	☐
Completing the exercise in this teaching method gave me a satisfying feeling of accomplishment.	☐	☐	☐	☐	☐
I learned somethings that were surprising or unexpected with this teaching method.	☐	☐	☐	☐	☐
**ATTITUDE: Affective Attitude Statement**					
The instructional approach increases my participation in the sexual health education.	☐	☐	☐	☐	☐
I feel happy to be taught sexual health education through this teaching method.	☐	☐	☐	☐	☐
The teaching method used makes sexual health education more interesting.	☐	☐	☐	☐	☐

Measure	SD	D	N	A	SA
The teaching method I attended is appropriate for the delivery of sexual health skills.	☐	☐	☐	☐	☐
The teaching method used is an ideal for sexual health education.	☐	☐	☐	☐	☐
**ATTITUDE: Cognitive Attitude Statement**					
The teaching method I attended enhanced my understanding of sexual health behaviour issues.	☐	☐	☐	☐	☐
The instructional approach explained the sexual health learning materials very well.	☐	☐	☐	☐	☐
I found the instructional worthwhile in the sexual health course.	☐	☐	☐	☐	☐
The method of instruction used in the sexual health course I attended was interesting.	☐	☐	☐	☐	☐
The teaching method used arouses interest in sexual health education program.	☐	☐	☐	☐	☐
**KNOWLEDGE: Importance of Knowledge Statement**					
The teaching approach enhanced my knowledge about STDs, STIs, and HIV/AIDS.	☐	☐	☐	☐	☐
I gained knowledge about reproductive health through this teaching method.	☐	☐	☐	☐	☐
I gained knowledge about sexual decision-making through the method of instruction.	☐	☐	☐	☐	☐
The instructional method used helped me gain knowledge about good manners and personal hygiene.	☐	☐	☐	☐	☐
**KNOWLEDGE: Application of Knowledge Statement**					
I will apply the sexual coercion and assault knowledge taught through this approach.	☐	☐	☐	☐	☐
The topic of responsible sexual behavioral practices taught in this approach seems very relevant.					
The method of teaching helped me gain knowledge on dealing with peer pressure during adolescence.	☐	☐	☐	☐	☐
**KNOWLEDGE: Effectiveness of Knowledge Statement**					
The method of instruction used is very active as it helped my understanding of the importance of abstinence.	☐	☐	☐	☐	☐
The teaching approach is very effective as it extended my existing understanding of the course.	☐	☐	☐	☐	☐
This teaching method is very effective in imparting knowledge in sexual health education programs.	☐	☐	☐	☐	☐
**ENGAGEMENT: Emotional Engagement Statement**					
The teaching method I attended it was very easy to understand the learning contents.	☐	☐	☐	☐	☐
I have been effective in this course as the method of instruction was engaging.	☐	☐	☐	☐	☐
The teaching method used facilitates my active	☐	☐	☐	☐	☐

Measure	SD	D	N	A	SA
participation in the subject taught.					
The method of instruction used caught my attention during the course.	☐	☐	☐	☐	☐
This method allowed my expression of thoughtful ideas relevant to the course.	☐	☐	☐	☐	☐
The instructional approach used during the course made me interested.	☐	☐	☐	☐	☐
**ENGAGEMENT: Cognitive Engagement Statement**					
I demonstrated my interest and enthusiasm as well as use of positive humor during the course.	☐	☐	☐	☐	☐
This teaching method is relevant for engaging students in the sexual education course.	☐	☐	☐	☐	☐
The teaching strategy used enhanced my engagement in the course.	☐	☐	☐	☐	☐
I focused on the learning activity given in this teaching approach.	☐	☐	☐	☐	☐

Notes:

- SD = Strongly Disagree (1 point);
- D = Disagree (2 points);
- N = Neutral (3 points);
- A = Agree (4 points); and
- SA = Strongly Agree (5 points).

This survey instrument was adapted from various prior studies[25–27] to reflect the research objectives.

References

32. **Sorbring E, Bolin A** and **Ryding J.** Game-Based - Intervention – A technical tool for social workers to combat adolescent dating violence. Adv Soc Work. 2015; 16(1): 125–139.
33. **Arnab S** and **Clarke S.** Towards a trans-disciplinary methodology for a game-based intervention development process. Br J Educ Technol. 2015; 48(2): 279–312. DOI: https://doi.org/10.1111/bjet.12377
34. **Evans AE, Edmundson-Drane E** and **Harris K.** Computer-assisted instruction: An effective instructional method for HIV prevention education. J Adolesc Heal. 2000; 26: 244–251. DOI: https://doi.org/10.1016/j.teln.2007.07.007
35. **Fitzpatrick VE.** Evaluability assessment of adolescent pregnancy prevention and sexual health - program, Be Proud! Be Responsible! In New York State [PhD Thesis]; 2015.
